# Concomitant detection of IFNα signature and activated monocyte/dendritic cell precursors in the peripheral blood of IFNα-treated subjects at early times after repeated local cytokine treatments

**DOI:** 10.1186/1479-5876-9-67

**Published:** 2011-05-17

**Authors:** Eleonora Aricò, Luciano Castiello, Francesca Urbani, Paola Rizza, Monica C Panelli, Ena Wang, Francesco M Marincola, Filippo Belardelli

**Affiliations:** 1 Department of Cell Biology and Neurosciences Istituto Superiore di Sanità, Rome, Italy; 2 Infectious Disease and Immunogenetics Section (IDIS), Department of Transfusion Medicine, Clinical Center and Trans-NIH Center for Human Immunology (CHI), National Institutes of Health, Bethesda, MD 20892, USA; 3 Cell Processing Section, Department of Transfusion Medicine, Clinical Center, National Institutes of Health, Bethesda, MD 20892, USA; 4 Scientific Affairs, Amgen Inc., Thousand Oaks, CA 91320-1799, USA

## Abstract

**Background:**

Interferons alpha (IFNα) are the cytokines most widely used in clinical medicine for the treatment of cancer and viral infections. Among the immunomodulatory activities possibly involved in their therapeutic efficacy, the importance of IFNα effects on dendritic cells (DC) differentiation and activation has been considered. Despite several studies exploiting microarray technology to characterize IFNα mechanisms of action, there is currently no *consensus *on the core signature of these cytokines in the peripheral blood of IFNα-treated individuals, as well as on the existence of blood genomic and proteomic markers of low-dose IFNα administered as a vaccine adjuvant.

**Methods:**

Gene profiling analysis with microarray was performed on PBMC isolated from melanoma patients and healthy individuals 24 hours after each repeated injection of low-dose IFNα, administered as vaccine adjuvant in two separate clinical trials. At the same time points, cytofluorimetric analysis was performed on CD14^+ ^monocytes, to detect the phenotypic modifications exerted by IFNα on antigen presenting cells precursors.

**Results:**

An IFNα signature was consistently observed in both clinical settings 24 hours after each repeated administration of the cytokine. The observed modulation was transient, and did not reach a steady state level refractory to further stimulations. The molecular signature observed *ex vivo *largely matched the one detected in CD14^+ ^monocytes exposed *in vitro *to IFNα, including the induction of CXCL10 at the transcriptional and protein level. Interestingly, IFNα *ex vivo *signature was paralleled by an increase in the percentage and expression of costimulatory molecules by circulating CD14^+^/CD16^+ ^monocytes, indicated as natural precursors of DC in response to danger signals.

**Conclusions:**

Our results provide new insights into the identification of a well defined molecular signature as biomarker of IFNα administered as immune adjuvants, and for the characterization of new molecular and cellular players, such as CXCL10 and CD14^+^/CD16^+ ^cells, mediating and possibly predicting patient response to these cytokines.

## Background

Interferons alpha (IFNα) are still the cytokines most widely used in clinical medicine today, with applications both in oncology and in the treatment of certain viral infections [1]. Several decades of research on IFNα have revealed that these cytokines exert immunomodulatory activities possibly involved in their *in vivo *therapeutic efficacy, spanning from the differentiation of the Th1 subset, the generation of CTL and the promotion of T cell *in vivo *proliferation and survival [reviewed in ref. [2]]. In particular, IFNα have proved to play an important role in the differentiation of monocytes into dendritic cells (DC) and in enhancing DC activities [3-8]. It has been suggested that IFNα-mediated DC activation can represent one of the mechanisms underlying the cytokine therapeutic efficacy *in vivo *[2].

In the attempt to understand in more detail the mechanisms of IFNα *in vivo*, several studies have recently utilized microarray technologies to detect and analyze an IFNα-specific signature in the peripheral blood cells of IFNα-treated individuals, with particular focus on HCV and melanoma patients [9-15]. These studies have revealed that many interferon-stimulated genes [16] (ISG), previously known to be induced by this cytokine in other animal or human *in vitro *settings, can be found up-regulated in the blood of patients treated *in vivo *with the cytokine. Furthermore, novel and unexpected ISG were added to the list of possible in *vivo *mediators of IFNα immunomodulatory and/or antitumor activity [9-15]. Defining with acceptable accuracy the pool of genes considered to be the signature of IFNα *in vivo *helps to understand the involvement of this cytokine in clinical as well as therapeutic settings [17,18]. Notably, an IFNα signature has been observed in systemic lupus erythematosus (SLE) patients, suggesting that the overexpression of a specific set of genes can represent the hallmark of *in vivo *cell exposure to IFNα, which is commonly detected in the sera of these patients [19]. More recently, the presence of a prominent IFNα signature has been reported in patients experiencing a growing list of autoimmune disorders, including psoriasis, multiple sclerosis, rheumatoid arthritis, dermatomyositis, primary biliary cirrhosis and insulin-dependent diabetes mellitus [20]. These data, together with the autoimmune-like phenomena reported in melanoma patients responding to IFNα therapy [21], confirmed the involvement of this cytokine in the delicate balance between immunity and autoimmunity.

Besides helping to gain insight into IFNα mechanisms of action *in vivo*, identifying a clear-cut IFNα signature *ex vivo *opens the possibility to define patterns of gene expression profiles significantly associated with IFNα treatment efficacy. In turn, this may also provide insights into candidate predictor biomarkers of response to therapy, and possibly assist in making the appropriate therapeutic decisions when a patient does not present with a favorable response profile. In spite of many efforts performed in this direction, the literature in this field suffers from a lack of consistency among the results obtained from patients suffering from different diseases and receiving different IFNα preparations. The majority of these studies have been performed in patients chronically infected with HCV, while attempting to identify a *consensus *blood biomarker predictive of IFNα/Ribavirin efficacy in patients blood [9-12,15]. Since it is known that the pattern of PBMC gene expression in HCV patients is altered by the infection itself [15], IFNα-induced modulations observed in these patients may be somehow related to the HCV disease, and possible affected by individual-specific variability, thus providing little information on the general mechanisms of action of the cytokine *per se*.

Despite the accumulating information on the IFNα-induced genes and of their possible *in vivo *role, little is known about the consistency of the IFNα signature in healthy *vs *cancer patients. A still elusive area of investigation is the kinetics of gene up-regulation in correlation with the possible appearance of immune cells elicited by IFNα and playing a primary role in the biological responses of IFNα-treated cancer patients. Likewise, no information is currently available on the transient and long-term effect of low doses of IFNα used with modalities typical of a vaccine adjuvant, as IFNα, in spite of their now recognized role as natural links between innate and adaptive immunity [2], have been extensively and generally used in clinics as typical antiviral or antitumor drugs. As a matter of fact, although the more effective and better tolerated pegylated IFNα2b is now widely used for the therapy of HCV infection [22] and in the adjuvant melanoma setting [23], no study is currently available on the clinical use of this molecule administered as vaccine adjuvant.

In the present study, we utilized PBMC derived from melanoma patients and healthy individuals, who had been enrolled in two clinical trials with similar treatment schedule, aimed at assessing the role of IFNα administered as vaccine adjuvant. We exploited microarray technology to evaluate and compare the modulations of PBMC global gene expression profiles induced by IFNα in melanoma and normal donors. The effects of the administration of different doses of IFNα, as well as of repeating the administration of the cytokine in successive treatment cycles, were evaluated. The kinetics and the biological significance of the modulations observed at the transcriptional level were correlated with the phenotypic changes observed in circulating CD14^+ ^and CD14^+^/CD16^+ ^monocytes. The overall results provide new insights in the identification of specific biomarkers for adjuvant IFNα and in the characterization of new molecular and cellular players mediating the response to this cytokine in patients.

## Methods

### Samples collection for gene profiling analysis from subjects receiving IFNα

PBMC for gene profiling analysis were obtained from patients enrolled in two studies sponsored by the Istituto Superiore di Sanità Rome, Italy. Both studies were approved by the Internal Review Board of the Istituto Superiore di Sanità and the clinical centers involved. Only subjects who have given informed written consent before initiating the trial were admitted to participate to the studies. In the first study, HLA-A*0201^+ ^stage IV metastatic melanoma patients underwent four cycles of vaccinations with gp100:209-217(210 M), IMDQVPFSV and Melan-A/MART-1 Melan-A/MART-1:26-35(27L), ELAGIGILTV melanoma peptides, given in combination with 3 million units (MU) of IFNα administered the previous day, in concomitance and the following day of the peptides inoculation [24]. The peptides were prepared under Good Manufacturing Practice conditions by Clinalfa (Laufelfingen, Switzerland) and were supplied as a water-soluble white powder in vials containing 250 μg of peptide. IFNα (human leukocyte IFNα; Alfaferone) was supplied by Alfawassermann (Bologna, Italy).

For gene profiling analysis on PBMC, blood was collected from six patients before any treatment (T0 and T42) and 24 hours after the IFNα plus peptide administration (T2 and T44). PBMC collections for gene profiling coincided with the first and the fourth vaccination (see Additional data file 1 for the complete treatment schedule).

For the second clinical study, healthy subjects previously unvaccinated against HBV were randomly divided into three groups to receive the HBV Engerix-B vaccine plus saline *placebo *or the HBV vaccine in association with human leukocyte IFNα (Alfaferone) at the dose of 1 or 3 MU [25]. Commercial pack of one monodose vial of Engerix-B (SmithKline Beecham), 20 μg/ml dose, was provided free of charge by Alfa Wassermann together with the IFNα and the *placebo *ampoules. The vaccination course was the standard 3-dose regimen administered at time zero (T0, baseline), one and six months later (T1 and T6m), in the *placebo *group, and two doses at T0 and T1m in the IFNα-treated groups. Blood samples were collected from 10 subjects per group for gene profiling analysis before (T0, T1m) and 24 hours after the *placebo *or IFNα plus vaccine administration (T0+24, T1m+24), and the collection was repeated on the first and the second cycle of vaccination (Additional data file 1).

The microarray data sets obtained from the two clinical trials were analyzed separately. For blood collection, 10 ml of peripheral blood was collected into BD vacutainer™ CPT™/sodium heparin tube and processed for the separation of mononuclear cells from whole blood according to the manufacturer's instruction. The recovered mononuclear cells were washed three times with PBS and resuspended in lysis buffer for RNA isolation (RNeasy, Qiagen).

### *In vitro *studies

PBMC were obtained by apheresis from 5 healthy donors at the Department of Transfusion Medicine, NIH. Total PBMC or the CD14^+ ^fraction (purity >98% as assessed by flow cytometry) isolated by column magnetic immunoselection (MACS Cell Isolation Kits; Miltenyi Biotec), were plated at the concentration of 2 × 10^6 ^cells/ml in OPTI-MEM medium (Gibco), and cultured at 37ºC and 5% CO_2 _in the presence of either IFNα2b (Intron A) or IFNγ1b (Actimmune) at the concentration of 1,000 U/ml. Cells were harvested and lysed in RLT buffer (Qiagen) and culture supernatants were collected for proteomic analysis 8 and 24 hours after stimulation respectively.

### RNA isolation and amplification and cDNA arrays

Total RNA was isolated using RNeasy mini kits (Qiagen). Amplified antisense RNA (aRNA) was prepared from total RNA (0.5-3 μg) according to a previously described protocol [26]. For hybridization to the microarrays, test samples were labeled with Cy5-dUTP (Amersham, Piscataway, NJ), and reference samples (pooled normal donor PBMC) were labeled with Cy3-UTP. Test-reference sample pairs were mixed and co-hybridized overnight to microarray slides in humidifying chambers. Test-reference sample pairs were mixed and co-hybridized to 17 K-cDNA microarrays. Microarrays were printed in house at the Immunogenetics Section, Department of Transfusion Medicine, Clinical Center, NIH, with a configuration previously described [27]. Hybridized arrays were scanned at 10-micrometer resolution on a Gene-Pix 4000 scanner (Axon Instruments, Downingtown, PA) at variable PMT voltage to obtain maximal signal intensities with less than 1% signal saturation. Resulting jpeg and data files were analyzed via mAdb Gateway Analysis tool [http://nciarray.nci.nih.gov]. The raw data set were filtered according to standard procedure to exclude spots with minimum intensity (arbitrarily set to < 200 in both fluorescence channels) or with diameters < 25 μm. Lowess intensity dependent normalization was used to adjust for differences in labeling intensities of the Cy3 and Cy5 dyes. The adjusting factor varied over intensity levels. All statistical analyses were done using the log2-based ratios. All analyses related to class comparison was done using the BRB-Array Tools [http://linus.nci.nih.gov/BRB-ArrayTools.html] developed by R. Simon et al [28]. Genes that were differentially expressed among the two classes were identified using a random-variance t-test [29]. Genes were considered statistically significant if their *p *value was < 0.001 and further analyzed by Cluster and Tree View software [30]. No adjustment was made for multiple comparisons. Gene annotations were mined using web-based tools such as DAVID [http://david.abcc.ncifcrf.gov/], GeneCards [http://www.genecards.org/index.shtml], COPE [http://www.copewithcytokines.de]. A modified Fisher Exact test was used for gene-enrichment analysis on Gene Ontology classification (by DAVID [http://david.abcc.ncifcrf.gov/]). Gene ratios are presented according to the central method for display [31].

### Quantitative PCR

QPCR was applied to detect the expression of BAFF, CXCL-10 and Mx transcripts using an ABI Prism 7900 HT (Applied Biosystems, Foster City, CA, USA). Primers and probes were custom-designed to span exon-intron junctions and generate < 150 base-pair amplicons (Biosource, Camarillo, CA, USA). Taqman probes were labeled at the 5' and 3' ends with the reporter FAM (6-carboxyfluorescein; emission λ_max _= 518 nm) and the quencher TAMRA (6-carboxytetramethylrhodamine; emission λ_max _= 582 nm), respectively. Standard curves were based on amplicons generated from PBMC exposed *in vitro *to IFNα2b (1,000 IU/ml); copy numbers were estimated with Oligo Calculator [http://www.pitt.edu/~rsup/OligoCalc.html]. Linear regression R^2^-values pertinent to all standard curves were ≥ 0.98. QPCR reactions were conducted in a 20 μl volume, including 1 μl cDNA, 1× Taqman Master MIX (Applied Biosystems), 2 μl of 20 μM primer and 1 μl of 12.5 μM probe. Thermal cycler parameters included 2 minutes at 50°C, 10 minutes at 95°C and 40 cycles involving denaturation at 95°C for 15 s, annealing-extension at 60°C for 1 minute. cDNA copy numbers were normalized according to the expression of Beta Actin as endogenous housekeeping gene [32].

### Cytofluorimetric analysis of monocytes *ex vivo*

For cytofluorimetric analysis, 30 ml of peripheral blood were collected into Vacutainer vials (Becton Dickinson) containing ACD as anticoagulant at each designated time point from donors enrolled in the HBV study. Blood was diluted 1:1 with sterile PBS then separated by Ficoll-Hypaque (Pharmacia,) density gradient to obtain PBMC. PBMC were washed twice, counted using Trypan Blue exclusion method, centrifuged again, resuspended at 30 × 10^6 ^cells/ml in 90% heat-inactivated foetal calf serum plus 10% DMSO and frozen in a -80°C freezer until shipment. Samples were shipped in dry ice. On arrival at the ISS, the vials were transferred into a liquid nitrogen tank. Samples of a single donor for each time-point (T0, T0+24, T1 and T1+24) were thawed and processed simultaneously. Number of viable cells was evaluated by trypan-blue exclusion method. 10 millions PBMC were incubated in presence of FcR Blocking Reagent (Miltenyi) to avoid not-specific staining, then treated with Dead Cell Discriminator Reagent (Miltenyi) and finally stained, in presence of Foetal Calf Serum and Sodium Azide, with fluorochrome-conjugated mAbs for 20 min at 4°C. The following mAbs were used: APC-conjugated anti-CD14, PE-conjugated anti-CD16, FITC-conjugated anti-HLA-DR (Becton Dickinson), FITC-conjugated anti-CD40 and anti-CD86 (Pharmingen). Samples were collected and analyzed by using a FACSCalibur (Becton Dickinson) and data analysis was performed by FlowJo software (Tree-Star), excluding dead cells and including cells falling in the expected morphological gate. The band pass filter used for cytofluorimetric analysis was 525 nanometers for FITC, 575 nanometers for PE and 675 nanometers for APC fluorochrome, respectively.

### Proteomic analysis on monocytes supernatants *ex vivo *and *in vitro*

After thawing of PBMC samples collected from donors enrolled in the HBV study, monocytes were derived from immunomagnetic selection and cultured *in vitro *at the concentration of 2 × 10^6 ^cells/ml in 2% human serum-AIMV medium alone or supplemented with HBsAg (10 μg/ml). 24 hours later, supernatants were collected and frozen immediately. The presence of CXCL-10 in the thawed supernatants was assessed by Searchlight Assay (Pierce-Endogen), consisting of a multiplex array measuring several proteins per well in standard 96-well plates where different monoclonal antibodies were spotted [33].

The same platform was used to detect the soluble factors released by monocytes isolated by healthy donors and exposed *in vitro *for 24 hours to 1,000 U/ml of IFNα2b (Intron A).

### Statistical analyses

Mann-Whitney and Wilcoxon Matched pairs nonparametric tests were used to investigate the significance of differences in specific PBMC populations between groups, as measured by citofluorimetry, for the proteomic analysis of monocytes supernatants and for Real Time PCR validation experiments.

## Results

### Signature of IFNα on human PBMC 24 hours after the cytokine administration

**Figure 1:**
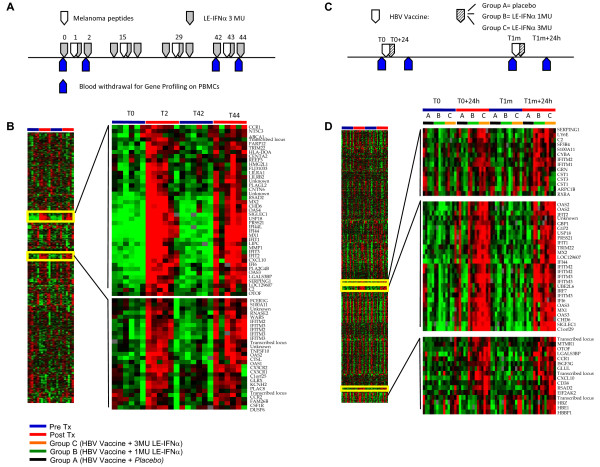
**Signature of IFNα on human PBMC 24 hours after the cytokine administration**. Treatment schedules for the clinical trials on melanoma patients (A) and healthy donors (C) examined in this study (see Methods and Additional data file 1). (B) Clusterogram showing the supervised hierarchical clustering of 6 PBMC samples collected before (T0, T42, blue bar) or after (T2, T44, red bar) the first and the fourth administration of IFNα plus melanoma peptides. The analysis was restricted to the 1093 most informative transcripts among the 17,000 of the complete dataset (80% gene presence across all experiments and at least 3-fold ratio change). (D) Clusterogram showing the supervised hierarchical clustering of all samples collected before (T0, T1m, blue bar) or after (T0+24, T1m+24, red bar) the first and the second cycle of administration of HBV vaccine in combination with *placebo *(black bar, group A), 1 (green bar, Group B) or 3 MU of IFNα (orange bar, Group C). The analysis was restricted 1712 most informative genes (see above). The enlargements show the nodes of genes specifically up-regulated after each IFNα plus vaccine administration.

As a first approach to analyze the data resulting from the microarray experiments on the PBMC isolated from melanoma patients (study 1, Figure 1A) and healthy subjects receiving HBV vaccine plus IFNα (study 2, Figure 1C), the two complete data sets profiling each 17,000 genes were independently filtered to sort out the most informative genes (80% gene presence across all experiments and at least 3-fold ratio change). Unsupervised hierarchical clustering obtained for the melanoma patients data set resulted in 1,093 genes, and did not segregate samples according to the IFNα plus peptide treatment (*data not shown*), suggesting that the majority of these transcripts were not dramatically affected by the cytokine administration *in vivo*. Conversely, when we performed a supervised hierarchical clustering analysis on this same set of 1,093 genes in (Figure 1C), grouping together samples collected at the time points analyzed, the visual inspection of the resulting clusterogram identified two nodes of genes showing a dramatic change in the level of expression after every peptides plus IFNα treatment. In particular, the expression of these genes was increased after the first administration of the cytokine, was back to the basal level 42 days later and increased again after the second IFNα administration. The IFNα-specific nodes, highlighted in Figure 1B-D, encompassed a signature of 68 transcripts, corresponding to 55 known and 6 unknown genes. Unsupervised hierarchical clustering analysis conducted on the data set of the second study (Study 2 on healthy subject) after filtering, resulted in 1,712 transcripts and in an characteristic signature cluster of 57 transcripts (corresponding to 47 known and 4 unknown genes) strongly up-regulated after every repeated administration of IFNα (Figure 1D). The comparison of the two IFNα-signature lists thus generated showed that 41 genes were up-regulated in both clinical settings 24 hours after the cytokine administration. This observation suggested that a signature of IFNα administration *in vivo *on human PBMC could be observed 24 hours after each consecutive cytokine administration and showed a similar kinetic trend in melanoma patients and healthy donors.

### Consistency of IFNα-induced modulation of PBMC gene expression profiles after each repeated administration of the cytokine

**Figure 2:**
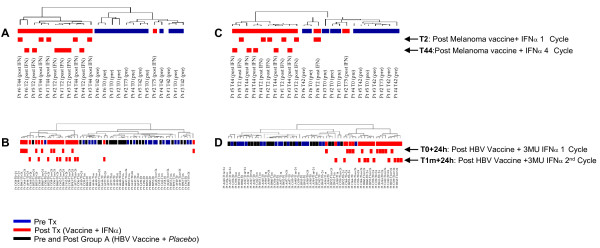
**Consistency of IFNα-induced modulation of PBMC gene expression profiles after each repeated administration of the cytokine**. Dendrogram showing the unsupervised hierarchical clustering of all samples available for gene profiling analysis from the melanoma and HBV study. For Melanoma study (A,B), the analysis was restricted to the 156 genes differentially expressed between T0 and T2 samples (A) or the 179 genes differentially expressed between T42 and T44 samples (B). For HBV study (C, D), the analysis was restricted to the 249 genes differentially expressed between T0 and T0+24 (C) of samples isolated from Group C patients (receiving 3MU IFNα), or to the 70 genes differentially expressed between T1m and T1m+24 (D) of the same group. Blue bar: "pre" IFNα plus vaccine samples; red bar: "post" IFNα plus vaccine samples; black bar: "pre" and "post" *placebo *plus vaccine samples.

We then moved to applying statistics to sort out the most informative genes from the whole database, and performed a class comparison analysis between the groups of PBMC samples collected before and after the treatment. For the study on melanoma patients, we initially focused on the first cycle of IFNα plus peptide administration. One hundred and fifty-six genes were significantly differentially expressed between T0 and T2 samples. Interestingly, when we let all samples available from this study (T0, T2, T42, T44) cluster according to the expression of these 156 genes, we obtained the segregation of all samples collected before (T0,T42) from samples collected after the treatment (T2, T44), regardless of the treatment cycle they belonged (Figure 2A). This result suggested that the modulation of PBMC global gene expression profile was consistently induced after each repeated administration of the cytokine, and was confirmed by reproducing the same phenomenon in PBMC obtained during the fourth therapeutic cycle of IFN administration in the melanoma study (Figure 2B). In fact, when we analyzed the transcripts of this second set of samples (T42, T44, collected 42 days after the beginning of the study and 24 hours after IFNα administration respectively), we found 179 genes differentially expressed between T42 and T44. Similarly to the transcripts profiling of cycle 1, the unsupervised hierarchical clustering of the complete database, based on the expression of these 179 genes obtained from cycle 4 class comparison analysis, not only segregated T42 from T44, but also separated T0 from T2 samples, with only a few samples behaving as outliers (Figure 2B).

A similar result was observed in the profiling of HBV specimens, for which a distinct segregation of all "pre" from all "post" IFNα plus vaccine samples (T0, T1m *vs *T0+24, T1m+24) could be obtained using either one of the gene expression sets (from cycle 1 or cycle 2) found to be significantly modulated by the HBV vaccine + 3MU of the cytokine (T0 *vs *T0+24 or T1m vs. T1m+24, Figure 2C-D). The same pattern of segregation was obtained for donors receiving 1MU of IFNα (*data not shown*)

### Similarity of the modulation of PBMC gene expression between two doses of IFNα tested

**Figure 3:**
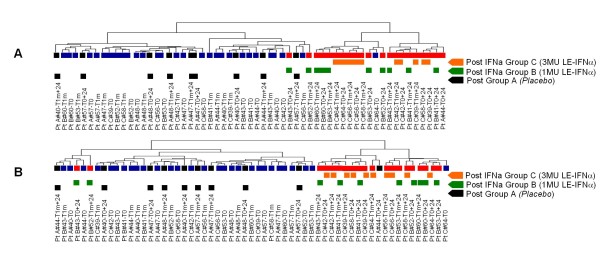
**Similarity of the modulation of PBMC gene expression between two doses of IFNα tested in combination with the HBV vaccine**. Dendrogram of the unsupervised hierarchical clustering analysis of all samples collected in the HBV study. In (A) the analysis was restricted to the expression of the 161 genes differentially expressed between all "pre" (T0, T1m) and all "post" (T0+24, T1m+24) samples isolated from healthy donors receiving HBV vaccine in combination with 3 MU of IFNα (orange bar, Group C). In (B), the same analysis was conducted on the 176 genes differentially expressed between all "pre" (T0, T1m) and all "post" (T0+24, T1m+24) samples isolated from donors receiving HBV vaccine plus 1 MU of LE-IFNα (green bar, Group B). Blue bar: "pre" IFNα plus vaccine samples; red bar: "post" IFNα plus vaccine samples; black bar: "post" *placebo *plus vaccine samples.

Taking advantage of the availability of blood samples obtained from patients receiving two different doses of IFNα, in the contest of the HBV study, we investigated whether the exposure to different doses of the cytokine caused a different modulation of PBMC gene expression. To address this issue, we selected the genes most consistently modulated by the cytokine by performing a class comparison analysis between all "pre" *vs *all "post" samples isolated from patients receiving 3 MU of IFNα. This class comparison identified 161 differentially expressed genes. Notably, the resulting gene list was not identical to the 176 gene list generated by comparing "pre" and "post" of samples isolated from patients receiving 1 MU of IFNα, since only 76 genes were overlapping (*data not shown*). However, hierarchical clustering of all samples from Group B (treated with 1 MU of IFNα) and C (treated with 3 MU of IFNα), restricted to the levels of expression of these 161 genes, showed that all "post" IFNα administration samples clustered together, whether they originated from patients receiving 1 or 3 MU of the cytokine (Figure 3A). The same result was obtained when the analysis was restricted to the 176 genes differentially expressed between all "pre" *vs *all "post" samples isolated from patients receiving 1 MU of IFNα (group B): this clustering segregated all "pre" from "post" samples, regardless of the dose of IFNα administered together with the vaccine (Figure 3B).

Taken together, these observations suggest that the two different doses of IFNα tested in our study gave rise to an extent of gene expression modulation that was somewhat similar. In particular, the trend of modulation achieved by the two doses of the cytokine was not close enough to generate identical gene lists after statistical analysis. However it was sufficiently similar to induce a similar change in PBMC gene expression, so that all "pre" and "post" samples were grouped together according to the intensity of expression of these genes, regardless of their original treatment group.

As expected, blood samples isolated from patients receiving *placebo *together with the HBV vaccine clustered together with the "pre" samples according to the expression of these both sets of IFNα-induced genes, confirming that these particular gene sets were more likely modulated by the cytokine and not by the vaccine itself (Figure 3).

### Genes up-regulated in the PBMC of humans receiving IFNα are mainly involved in immune response-related functions

**Figure 4:**
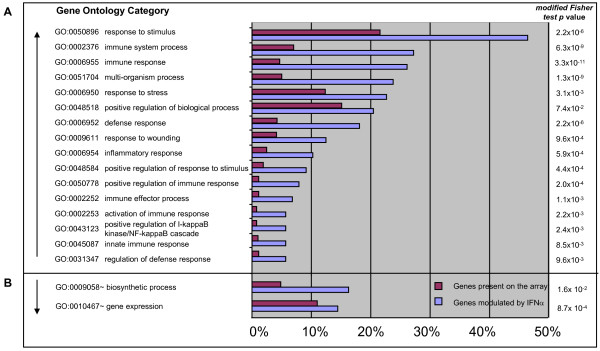
**Functional classification of genes modulated by IFNα *in vivo***. The list of transcripts significantly modulated by IFNα *in vivo *was selected by matching the lists generated by Class comparison between all "pre" *vs *"post" treatment samples in melanoma patients and healthy subjects receiving IFNα as vaccine adjuvant (487 and 311 respectively). The gene lists were matched, and the 130 genes consistently up-regulated (A) and the 34 consistently down-regulated (B) were separately analyzed for Gene Ontology by means of David (Biological Process, ALL levels). Biological process classes were ranked according to the percentage of the genes of the lists fitting each class (blue) in proportion to the global composition of the array (light purple). The *p *value of the modified Fisher test classes enrichment (*p *< 0.05) is shown.

In order to gain insights into the mechanisms of action of IFNα administered *in vivo*, we performed the functional classification (based on Gene Ontology) of genes found to be up-regulated or down-regulated by the cytokine in human PBMC. In particular, the most consistently modulated genes were selected by matching the gene list obtained by the class comparison of all "pre" and "post" IFNα administration samples in melanoma patients (T0-T42 *vs *T2-T44, yielding to 311 genes) *with that of *healthy donors vaccinated with HBV plus IFNα (T0-T1m samples from groups B and C grouped together *vs *T0+24h-T1m+24h samples from the same groups, yielding to 487 genes). Figure 4 shows the biological process classes of the *in vivo *IFNα modulated genes, ranked according to the enrichment level of each class and compared to the global composition of the array (modified Fisher test *p *< 0.05). The blue bar represents the percentage of genes modulated by IFNα belonging to each specific Gene Ontology category, and the purple bar corresponds to the percentage of genes represented on the array assigned to the same Gene Ontology category. The results of this classification showed that the most represented classes of the 130 up-regulated transcripts, included genes involved in the response to virus or external stimuli, immune-related genes, or genes involved in the inflammation process (Figure 4A). The IFNα-induced modulation of some of these genes, such as CXCL10, BAFF and Mx, was confirmed by real time PCR (Additional data file 2).

Interestingly, a much lower level of consistency was observed for the genes found to be down-regulated by IFNα *in vivo *(Figure 4B), since for this category only 34 genes, mostly associate with general biosynthetic process or gene expression Gene Ontology Categories, were found to be in common between the two studies.

### Consistency of IFNα signature in different *in vivo *and *in vitro *settings

**Figure 5:**
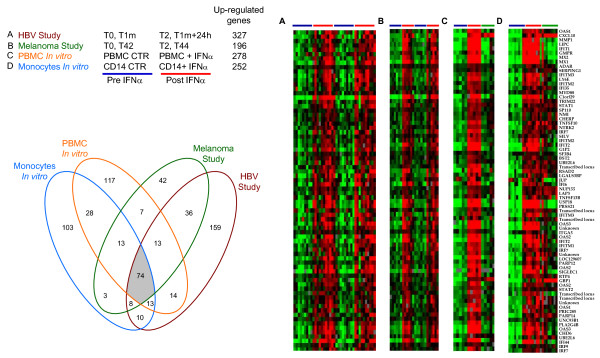
**Consistency of IFNα signature in different *in vivo *and *in vitro *settings**. Heatmap of the 74 cDNA consistently up-regulated by IFNα in any of the *in vivo *and *in vitro *settings analyzed. The lists of IFNα-up-regulated genes differentially expressed between all "pre" and "post" samples in healthy donors receiving HBV vaccine plus IFNα (A), all "pre" and "post" samples in Melanoma patients vaccinated with melanoma peptides plus IFNα (B), total PBMC (C) and CD14^+ ^monocytes (D) isolated from healthy donors and untreated or treated with IFNα2b *in vitro *were matched (see Venn diagram), and the expression of the 74 genes in common among all lists was visualized in each of the databases as separate heatmap. Blue bar: "pre" IFNα plus peptide or vaccine samples; red bar: "post" IFNα *in vitro *(C, D) or vaccine plus IFNα *in vivo *samples (A, B); green bar: "post" IFNγ *in vitro *samples (D).

In the attempt to unravel the "core" signature of IFNα, representative of the effect of this cytokine *in vivo *as well as *in vitro*, we compared our microarray data on PBMC obtained from subjects receiving IFNα *in vivo *(study 1 and 2) with the profiling of transcripts expressed by PBMC and monocytes exposed to IFNα *in vitro*. To this end, total PBMC as well as purified CD14^+ ^monocytes isolated from five healthy donors were incubated *in vitro *with IFNα2b, IFNγ to control for IFNα specific effects (10^3 ^IU/ml) or no stimulus for eight hours. For ethical reasons, we could not collect more blood samples from subjects enrolled in the clinical studies to perform the *in vitro *study. We first performed a comparison among the transcripts of *in vitro *treated PBMC, monocytes and respective controls. This analysis resulted in a set of 376 transcripts for PBMC exposed to IFNα (278 up-regulated and 98 down-regulated) and 304 transcripts for IFNα-treated monocytes (252 up-regulated and 52 down-regulated). This gene lists were then compared with the two lists of genes previously found to be modulated by the cytokine in the two clinical studies examined (all "pre" versus "post-IFNα" samples for melanoma patients, yielding to 196 up-regulated genes, and healthy subjects vaccinated with 1 or 3 MU of IFNα, yielding to 327 up-regulated genes). The comparison of these 4 gene lists resulted in 74 transcripts corresponding to 64 genes consistently up-regulated after exposure to IFNα in all the settings examined (Figure 5). Interestingly, although a few of these genes showed a trend of increase also in monocytes and PBMC exposed *in vitro *to IFNγ, the induction was much stronger in terms of log ratio levels and more consistent among groups of samples treated with IFNα, confirming that these 64 genes can be considered the IFNα specific signature in human PBMC as well as monocytes.

### Enhanced transient expression of costimulatory molecules and HLA-DR in CD14^+ ^and CD14^+^/CD16^+ ^monocytes after acute exposure to IFNα in healthy individuals

**Figure 6:**
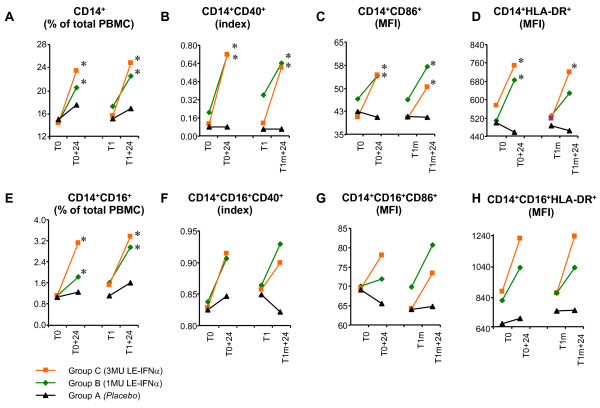
**Immunophenotipic analysis of PBMC of subjects treated with IFNα**. Effects of IFNα administration on PBMC subsets phenotype *in vivo*, analyzed by FACS analysis on PBMC obtained from healthy donors undergoing HBV vaccine plus IFNα administration. Blood samples were drawn before (T0, T1m) and 24 h after (T0+24, T1m+24) the first and the second vaccine administration and cells were isolated by ficoll gradient-centrifugation and labeled with specific antibodies, as described in Methods section. For each subset, the most appropriate parameter (% of total PBMC; index = CD14^+^CD40^+^/CD14^+^CD40^- ^or CD14^+^CD16^+^CD40^+^/CD14^+^CD16^+^CD40^- ^ratio; MFI = mean fluorescence intensity) is shown for the average of n = 10 samples per group. * *p *< 0.05 (Wilcoxon Matched Pairs test).

In order to further evaluate the effects of IFNα administration *in vivo*, with particular focus on antigen presenting cell precursors, an immunophenotypic analysis was performed by multicolor flow cytometry on PBMC obtained from healthy subjects before and shortly after HBV vaccine and IFNα administration. The results, shown in Figure 6, provided evidence that at 24 hours after the treatment the percentage of CD14^+ ^monocytes in the whole PBMC populations was significantly higher in both IFNα-treated groups (1 or 3 MU) (Figure 6A). The IFNα administration also resulted in a significant transient increase of the percentage of CD14^+ ^monocytes expressing the costimulatory molecule CD40 as compared to the administration of *placebo *(Figure 6B). Monocytes isolated from IFNα-treated donors were also endowed with a CD86 showing a higher mean fluorescence intensity (Figure 6C) and with a trend of increase of the expression of HLA-DR (Figure 6D). No significant increase of these molecules was detected by cytofluorimetric analysis in the CD14^+ ^monocytes isolated from the group of subjects receiving *placebo *together with the HBV vaccine (Figure 6A-D).

A similar effect was found in cells expressing both CD14 and CD16, reported to be more mature than CD14 and showing features of tissue macrophages [34]. In particular, the percentage of CD14^+^/CD16^+ ^among total donors PBMC increased after the first IFNα administration was found back to basal level one month later and rose again after the second treatment (Figure 6E). Also for these cells, the analysis showed an increased expression of costimulatory molecules CD40 and CD86 and of HLADR after each IFNα administration (Figure 6F-H).

### Release of chemotactic chemokines by monocytes isolated from subjects exposed to IFNα

**Figure 7:**
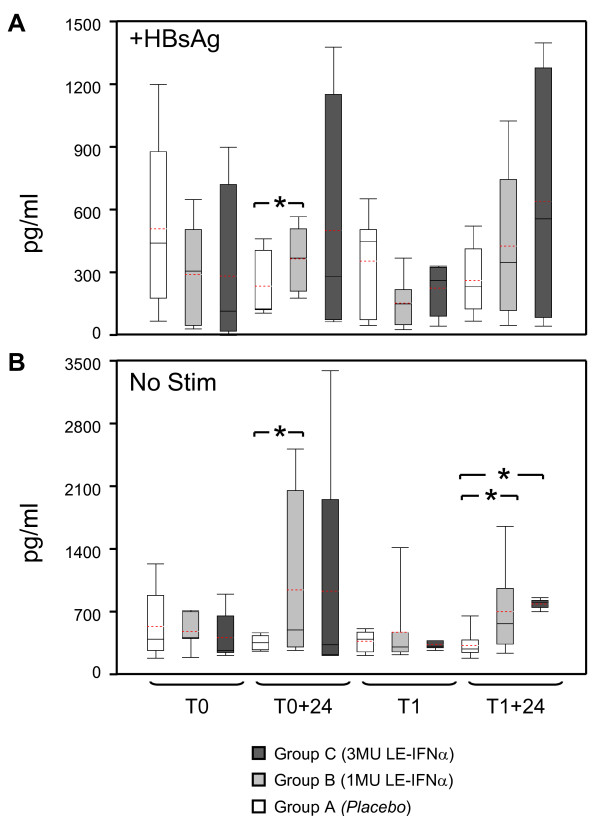
**Release of CXCL-10 by CD14+ monocytes exposed to IFNα *in vivo***. Release of CXCL-10 by CD14^+ ^monocytes exposed to IFNα *in vivo*. The levels of CXCL-10 were measured in the supernatants of monocytes isolated from PBMC collected 24 hours after treatment from subjects receiving *placebo *(white bar), 1 (grey bars) or 3 (black bars) MU of IFNα + HBV vaccine. Monocytes isolated from 5-6 subjects per group were sensitized *in vitro *with the HBV specific antigen HBsAg (A) or left untreated (B) as described in Methods section. Red dotted line: Mean, Black line: Median, Box: 25^th ^to 75^th ^percentile, whiskers:10^th ^to 90^th ^percentile. * *p *< 0.05 (Wilcoxon Matched Pairs test).

The induction of CXCL10 by IFNα, observed at a molecular level by microarray analysis on PBMC, was further investigated by performing a proteomic assay on supernatants of CD14^+ ^monocytes isolated from healthy donors receiving the cytokine in association with the HBV vaccine. The results, reported in Figure 7, showed that monocytes collected 24 hours after the administration of IFNα (1 or 3 MU) either sensitized *in vitro *with the HBV specific antigen HBsAg (Figure 7A) or left untreated as control (Figure 7B) had an increased ability to release CXCL10 in the culture supernatants as compared to pre-treatment samples or to samples of donors receiving *placebo*. In particular, the kinetic of CXCL10 release during the first and second cycle of vaccination resembled the trend of induction observed at the mRNA level, with the samples collected one month after vaccination showing basal levels of CXCL10, and a considerable raise 24 hours after each cytokine administration.

The pattern of soluble factors released by human blood cells in response to IFNα was also evaluated in the *in vitro *model of monocytes isolated from healthy donors PBMC and exposed *in vitro *to IFNα2b, where the significant release of CXCL10 was also observed, together with the production of other 5 chemokines (Additional data file 3).

## Discussion

In the study presented herein, we applied microarray technology to profile the gene expression in human PBMC treated *in vivo *with IFNα, administered at low dose as vaccine adjuvant in the context of two separate clinical trials, performed on melanoma patients and healthy subjects, following a similar treatment schedule. A clear-cut signature of IFNα *in vivo *could be observed in human PBMC 24 hours after the cytokine administration in both clinical studies (Figure 1). Interestingly, the modulation of PBMC global gene expression profile was consistently induced after each repeated administration of the cytokine, suggesting that, at least at the transcriptional level, the extent of the modulations induced by the cytokine is mainly transient, and does not reach a steady state level refractory to further stimulations (Figure 2). In general, the transcriptional modulations observed appear quite homogeneous among the different subjects analyzed, and no major differences between groups of subjects receiving two different doses of the cytokine were observed (Figure 3).

The results of our transcriptional profiling provided the molecular basis supporting a predominant immunomodulatory role of IFNα when administered as vaccine adjuvant. According to the gene ontology analysis (Figure 4), the immunological pathways influenced by IFNα *in vivo *recapitulate the progression of the main steps for the generation of a specific immune response, from the early non specific antiviral defense (OAS, MX), to inflammation (TLR7, NMI, CXCL10, MYD88), recruitment of immune cells (CXCL10, C3AR1, CX3CR1), antigen processing and presentation (PSMB9, HLA-DOA) and finally to the effectors specific immune response: (SERPING1, C2, BST2, MYD88, TNFSF13B/BAFF).

Since PBMC is a heterogeneous population consisting of various subsets of cells that may experience different responses to IFNα, changes at the transcript level observed in total PBMC specimens cannot be ascribed to a specific immune effect. However, when we compared human PBMC isolated after the *in vivo *administration of the cytokine, to PBMC or purified monocytes isolated from healthy donors and exposed *in vitro *to the cytokine, we found a significant correlation among the IFNα-up-modulated genes in the various group, so that we were able to define a "core" IFNα signature consistently observed in all the *in vivo *and *in vitro *settings (Figure 5). Interestingly, among the genes consistently up-regulated by IFNα associated with inflammation, we found the metalloprotease MMP-1 not previously reported to be an ISG (to the best of our knowledge), and involved in extra cellular matrix degradation for cells migration and tissue remodeling, during physiological and pathological conditions [35]. Of interest, MMP-1 can be released by macrophages, monocytes [35] and monocyte-derived DC [36], and alteration of its expression has been recently associated with autoimmunity phenomena [36,37]. The "core" signature of IFNα identified in our *in vitro *and *in vivo *experiments also included BAFF, a gene showing a crucial role in B cell maturation and activity, reported to be involved in the pathogenesis of autoimmune diseases, such as Rheumatoid arthritis, SLE and Progressive Systemic Sclerosis in mouse models as well as in humans [38]. Moreover, our list included at least 4 genes belonging to TLR7 pathway (Myd88, IRF7, CXCL10 and STAT1), a system responsible for the activation of the innate immune response in response to RNA viruses, but also implicated in IFNα-related autoimmune phenomena, mainly through plasmacytoid DC [39]. Interestingly, TLR7 have been reported to be expressed by IFN-DC, which could also secrete IFNα following viral stimulation or TLR7-specific stimulation, thus confirming the critical role of this cytokine at the early steps of immune response to pathogens or in autoimmune diseases [8].

Of interest, although a rigorous comparison among the results of different microarray studies is impaired by the bias possibly induced by different platforms and statistical approaches, the core IFNα signature identified by us in subjects receiving the cytokine as vaccine adjuvant is not considerably different, in terms of modulated genes and Gene Ontology categories, from the one reported by studies investigating the same issue in HCV-infected patients treated with IFN (IFNα2b or PEG-IFNα2b) and Ribavirin [9-16]. Moreover, our data on the transcriptional modulations observed in PBMC treated *in vitro *with IFNα2b are concordant with data reported by others on the effects on the same cells of the pegylated form of the cytokine administered in association with Ribavirin [40]. Overall, these observations strongly suggest that a similar signature occurs both *in vivo *and *in vitro *(at least in PBMC), regardless of the dose or type of IFNα used or even of the condition of the subjects receiving the cytokine (healthy donors and HCV-infected or cancer patients).

The proteomic analysis of the supernatants of monocytes exposed *in vitro *to IFNα (Additional data file 3) confirmed at the protein level the effect of this cytokine on chemoattraction and inflammation observed at the transcription level *in vitro *and *in vivo*, corroborating the results of the gene ontology analysis on the immunomodulatory role of IFNα *in vivo*. Although further studies on specific cell subsets isolated *ex vivo *from subjects receiving IFNα are needed to define the role of monocytes in the cytokine activity *in vivo*, our results suggest that monocytes contribute to the transcriptional modulation seen on total PBMC, in line with previous observations from our group and others on IFNα linking innate and adaptive immunity by affecting monocytes differentiation into DC [reviewed in ref. [2]].

To gain more insight into the specific effects of IFNα on the several monocytes blood populations, we analyzed the immunophenotype changes observed in PBMC isolated from healthy donors before and after IFNα administration, with particular focus on CD14^+ ^cells. Of note, at the same time of detection of the typical IFNα signature in PBMC, we also observed an increase in the percentage of CD14^+ ^and CD14^+^/CD16^+ ^monocytes, and both cell populations proved to express high levels of costimulatory molecules and HLA-DR (Figure 6). Notably, such increase was transient, similarly to the appearance of the IFNα signature, and additional rounds of increase were observed at 24 hr after the subsequent IFNα treatments, in parallel with the *de novo *detection of an up-regulated expression of the typical IFNα-induced genes.

CD14^+^/CD16^+ ^monocytes, coexpressing CD16 and low levels of CD14, were first characterized by Ziegler-Heitbrock and colleagues in 1988 [41], and their number and phenotype/function have been reported to be altered in patients with cancer, infectious diseases or inflammatory disorders [42-45]. Of note, an increase of CD14^+^/CD16^+ ^monocytes was observed in patients infected with pathogens triggering IFNα production, such as certain bacteria and HIV [45]. In general, this cell subset has been indicated as a transitional stage of development of monocytes to macrophages, originating from CD14^high^CD16^+^, or DC, derived from CD14^dim^CD16^+ ^cells [46], and has been shown to exhibit special capabilities to migrate [47], to stimulate CD4^+ ^T cells [48] and to produce proinflammatory cytokines [45]. Moreover, CD16^+ ^monocytes can differentiate in CD1b^+ ^DC endowed with high APC capacity after a short time exposure to TLR2 ligands [49], supporting the concept that these cells may represent natural precursors of DC in response to danger signals. In the light of all this, it is possible that the transient up-regulation of costimulatory molecules and HLA-DR in CD16^+ ^monocytes, occurring at the time of the appearance of a PBMC IFNα molecular signature, characterized by enhanced expression of immune-related cytokines/chemokines, can represent a reliable marker of the biologic response to a local IFNα treatment, which may result in the generation of active DC, resembling those naturally generated from this monocyte subset in response to infections and danger signals. Notably, an ensemble of studies from our group and from other laboratories have demonstrated that IFNα can induce a very rapid differentiation of highly active DC from monocytes [50] and these DC (IFN-DC) are characterized by a special signature [51], which partially overlaps with the IFNα-signature described in the present study. In this regard, it is worth mentioning recent results indicating that spontaneous regression of highly immunogenic *Molluscum contagiosum *virus-induced skin lesions is associated with the infiltration of DC strongly resembling IFN-DC [52], supporting the concept that IFN-DC can indeed represent naturally occurring DC promptly generated *in vivo *during the response to type I IFN induced by viruses and other natural danger signals.

Of interest, a recent study by Mohty and colleagues [53] has shown the increase of CXCL-10 plasma levels in melanoma patients treated with relatively low doses of IFNα, which also parallels a trend towards an increase of CD16^+ ^monocytes. CXCL10 is an IFNα-induced chemokine, which binds and activates the seven transmenbrane G-protein-coupled receptor CXCR3, and is expressed especially in activated Th1 cells, B cells, NK cells and DC, thus suggesting that CXCL10 release can represent a primary event in the amplification of the IFNα response. The results of Mohty and coworkers [53] are consistent with our data showing an up-regulation of CXCL10 expression after local low-dose IFNα injection, as revealed by both genomic and proteomic analysis *ex vi*vo and *in vitro*. Of note, the up-regulation of CXCL-10 has been reported to occur also in HCV-infected patients shortly after the administration of PEG-IFNα2b [8], so that it has been suggested that CXCL-10 can represent a marker predictive of the final treatment outcome [54].

## Conclusion

Overall, the results presented herein show that: i) the production of CXCL10 and a specific IFNα-signature are observed in PBMC as early as 24 hr after cytokine injection in healthy donors and melanoma patients; ii) such response is transient, does not reach a steady-state level of refractoriness and can occur after inoculation of as little as 1 millions of units of IFNα. iii) The observed molecular signature is paralleled by the raise in percentage and expression of costimulatory molecules of CD14^+^/CD16^+ ^peripheral blood cells, reported to be precursors of DC in response to danger signals. These results shed a new light on the immune mechanisms of action of IFNα and, in particular, on the role of CXCL10 and early effects of IFNα on monocyte/DC precursors (such as CD14^+^/CD16^+ ^cells) as primary players in the IFNα response and stimulate further studies for identifying molecular and biological markers capable of predicting the clinical response to IFNα.

## List of Abbreviations

IFN: Interferon; DC: Dendritic Cells; PBMC: Peripheral Blood Mononuclear Cells; Th: T helper; CTL: Cytotoxic T Lymphocytes; HCV: Hepatitis C Virus; ISG: Interferon Stimulated Genes.

## Competing interests

The authors declare that they have no competing interests.

## Authors' contributions

EA performed all microarray experiments *ex vivo *and *in vitro*, including Real Time PCR validation, carried out all data analysis and wrote the paper; LC performed microarray data analysis and contributed to writing the paper; FU performed cytofuorimetric and proteomic analysis on samples isolated from the HBV clinical study; PR planned and organized the HBV clinical study; EW and MCP supervised the microarray experiments and data analysis; FMM designed and overall supervised the microarray experiments; FB designed and supervised the entire research and revised the paper. All authors read and approved the final manuscript.

## Supplementary Material

Additional file 1 **Complete treatment schedule of the clinical studies examined and blood samples collection for gene profiling analysis**. (A) HLA-A*0201^+ ^stage IV metastatic melanoma patients underwent four cycles of vaccinations with gp100:209-217(210M), IMDQVPFSV and Melan-A/MART-1 Melan-A/MART-1:26-35(27L), ELAGIGILTV melanoma peptides (white arrows), given in combination with 3 MU of IFNα (grey arrows) administered the previous day, in concomitance and the following day of the peptides inoculation. For gene profiling analysis on PBMC, blood was collected before (T0 and T42) and 24 hours after the IFNα plus peptide administration (T2 and T44) (Blue arrows). PBMC collections for gene profiling coincided with the first and the fourth vaccination. (B) Healthy subjects were randomly divided into three groups to receive the HBV Engerix-B vaccine (white arrows) plus saline *placebo *or the HBV vaccine (grey arrows) in association with human leukocyte IFNα (Alfaferone) at the dose of 1 or 3 MU. The vaccination course was the standard 3-dose regimen administered at time zero (T0, baseline), one and six months later (T1 and T6m), in the *placebo *group, and two doses at T0 and T1m in the IFNα-treated groups. For gene profiling analysis on PBMC, blood samples were collected from 10 subjects per group before (T0, T1m) and 24 hours after the *placebo *or IFNα plus vaccine administration (T0+24, T1m+24), and the collection was repeated on the first and the second cycle of vaccination. Click here for file

Additional file 2 **Real time PCR validation of microarray data**. Real Time PCR validation of the expression of BAFF (A, B), CXCL10 (C, D) and Mx (E, F) transcripts in samples collected at different time points during the melanoma (a, c, e) and HBV (b, d, f) studies. The box plot graph shows cDNA copies for each gene, normalized by the copies of Beta Actin as housekeeping, measured for five samples per group. Red line: Mean, Black line: Median, Box: 25^th ^to 75^th ^percentile, whiskers:10^th ^to 90^th ^percentile. * *p *< 0.05 (Wilcoxon Matched Pairs test). Click here for file

Additional file 3 **Release of chemotactic chemokines by CD14^+ ^monocytes exposed to IFNα *in vitro***. Chemotactic chemokines released by monocytes isolated from healthy donors and exposed *in vitro *to 10^3^IU/ml of IFNα. The graph shows the 6 factors selected, out of a panel of 46 tested, for being significantly enriched in the supernatants of cells exposed to the cytokine as compared to untreated controls. The box plot graph shows for 10 samples per group: Red line: Mean, Black line: Median, Box: 25^th ^to 75^th ^percentile, whiskers:10^th ^to 90^th ^percentile. *p < 0,005, ^§^*p *< 0,05 (Wilcoxon Matched Pairs test). Click here for file
