# Supplementary material for: Concomitant detection of IFNα signature and activated monocyte/dendritic cell precursors in the peripheral blood of IFNα-treated subjects at early times after repeated local cytokine treatments
Source: J Transl Med. 2011 May 17;9:67. doi: 10.1186/1479-5876-9-67 (PMC3115876; doi:10.1186/1479-5876-9-67)
Supplement: Additional file 1 — Complete treatment schedule of the clinical studies examined and blood samples collection for gene profiling analysis. (A) HLA-A*0201+ stage IV metastatic melanoma patients underwent four cycles of vaccinations with gp100:209-217(210M), IMDQVPFSV and Melan-A/MART-1 Melan-A/MART-1:26-35(27L), ELAGIGILTV melanoma peptides (white arrows), given in combination with 3 MU of IFNα (grey arrows) administered the previous day, in concomitance and the following day of the peptides inoculation. For gene profiling analysis on PBMC, blood was collected before (T0 and T42) and 24 hours after the IFNα plus peptide administration (T2 and T44) (Blue arrows). PBMC collections for gene profiling coincided with the first and the fourth vaccination. (B) Healthy subjects were randomly divided into three groups to receive the HBV Engerix-B vaccine (white arrows) plus saline placebo or the HBV vaccine (grey arrows) in association with human leukocyte IFNα (Alfaferone) at the dose of 1 or 3 MU. The vaccination course was the standard 3-dose regimen administered at time zero (T0, baseline), one and six months later (T1 and T6m), in the placebo group, and two doses at T0 and T1m in the IFNα-treated groups. For gene profiling analysis on PBMC, blood samples were collected from 10 subjects per group before (T0, T1m) and 24 hours after the placebo or IFNα plus vaccine administration (T0+24, T1m+24), and the collection was repeated on the first and the second cycle of vaccination. [file 1479-5876-9-67-S1.PPT]

## Slide 1
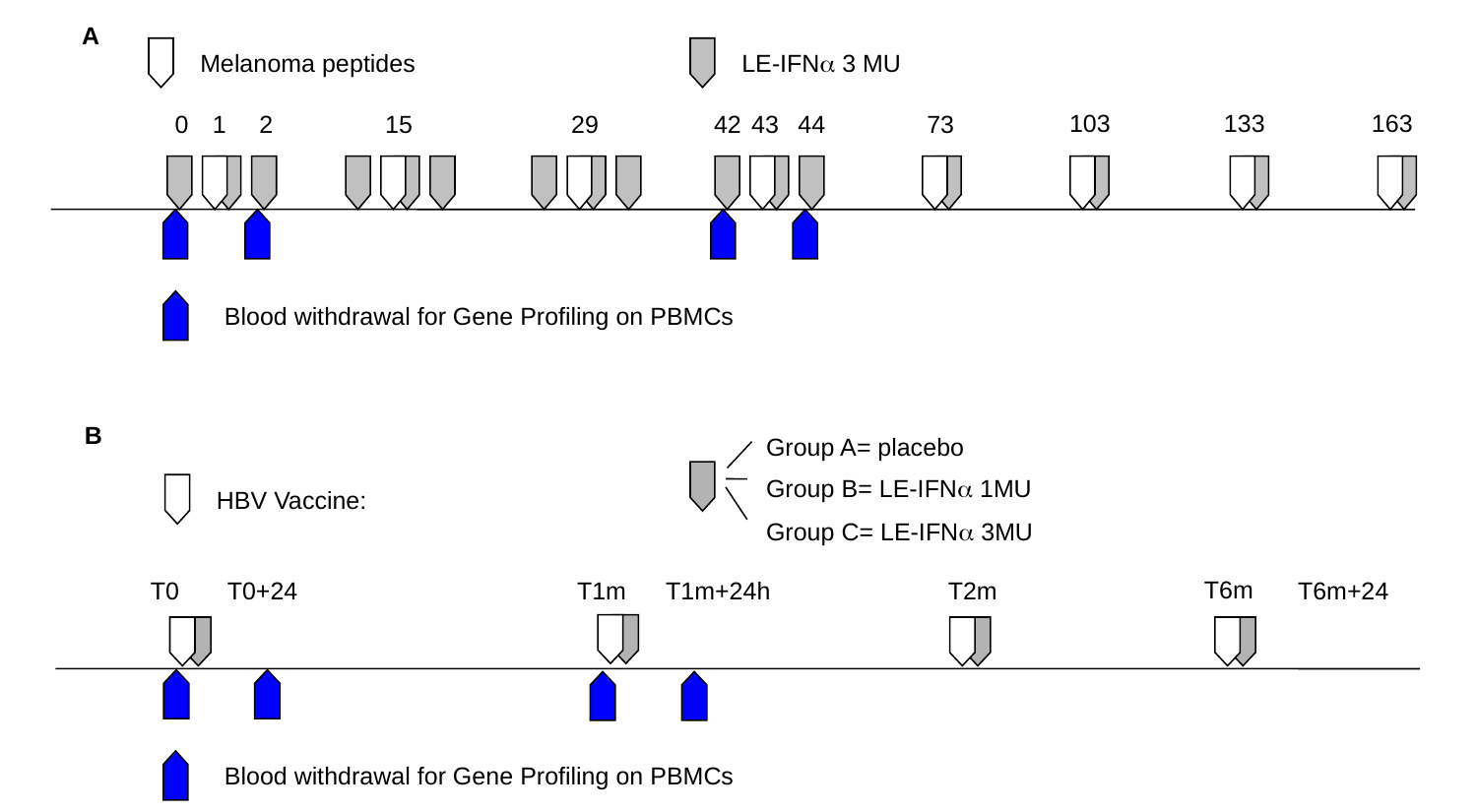

A
LE-IFNα 3 MU
Melanoma peptides
0
1
2
15
29
42
43
44
73
103
133
163
Blood withdrawal for Gene Profiling on PBMCs
B
Group A= placebo
Group B= LE-IFNα 1MU
Group C= LE-IFNα 3MU
HBV Vaccine:
 T0
 T0+24
 T1m
T1m+24h
T2m
T6m
T6m+24
Blood withdrawal for Gene Profiling on PBMCs
