# Supplementary material for: Concomitant detection of IFNα signature and activated monocyte/dendritic cell precursors in the peripheral blood of IFNα-treated subjects at early times after repeated local cytokine treatments
Source: J Transl Med. 2011 May 17;9:67. doi: 10.1186/1479-5876-9-67 (PMC3115876; doi:10.1186/1479-5876-9-67)
Supplement: Additional file 2 — Real time PCR validation of microarray data. Real Time PCR validation of the expression of BAFF (A, B), CXCL10 (C, D) and Mx (E, F) transcripts in samples collected at different time points during the melanoma (a, c, e) and HBV (b, d, f) studies. The box plot graph shows cDNA copies for each gene, normalized by the copies of Beta Actin as housekeeping, measured for five samples per group. Red line: Mean, Black line: Median, Box: 25th to 75th percentile, whiskers:10th to 90th percentile. * p < 0.05 (Wilcoxon Matched Pairs test). [file 1479-5876-9-67-S2.PPT]

## Slide 1
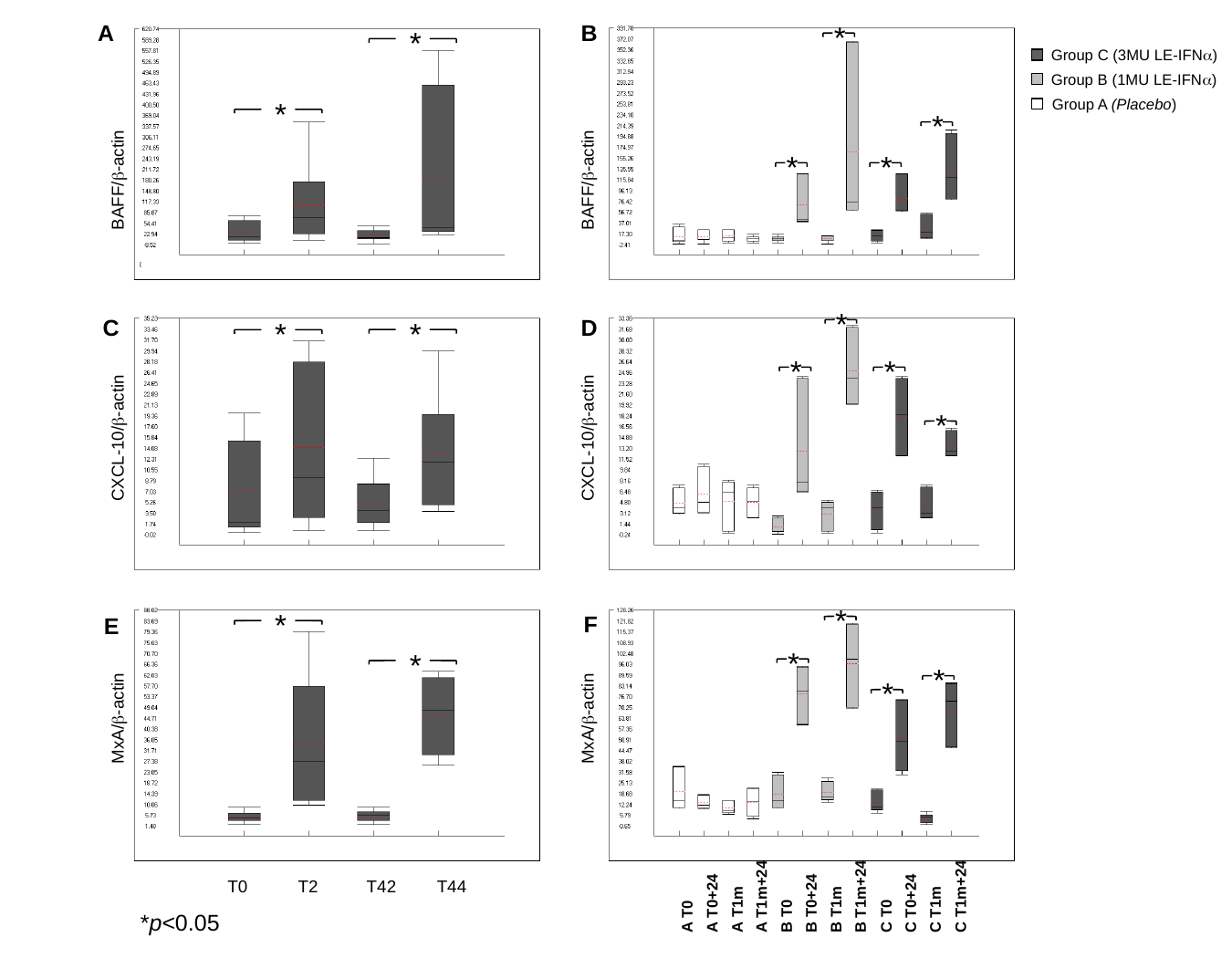

A
B
BAFF/β-actin
D
CXCL-10/β-actin
B T1m+24
A T1m+24
C T1m+24
A T1m
A T0+24
B T0+24
B T1m
C T0+24
C T1m
A T0
B T0
C T0
F
MxA/β-actin
BAFF/β-actin
C
CXCL-10/β-actin
T0
T2
T42
T44
E
MxA/β-actin
*
*
*
*
*
Group C (3MU LE-IFNα)
Group B (1MU LE-IFNα)
Group A (Placebo)
*
*
*
*
*
*
*
*
*
*
*
*
*
*p<0.05
