# Supplementary figures and images for: Concomitant detection of IFNα signature and activated monocyte/dendritic cell precursors in the peripheral blood of IFNα-treated subjects at early times after repeated local cytokine treatments
Source: J Transl Med. 2011 May 17;9:67. doi: 10.1186/1479-5876-9-67 (PMC3115876; doi:10.1186/1479-5876-9-67)

## Slide 1
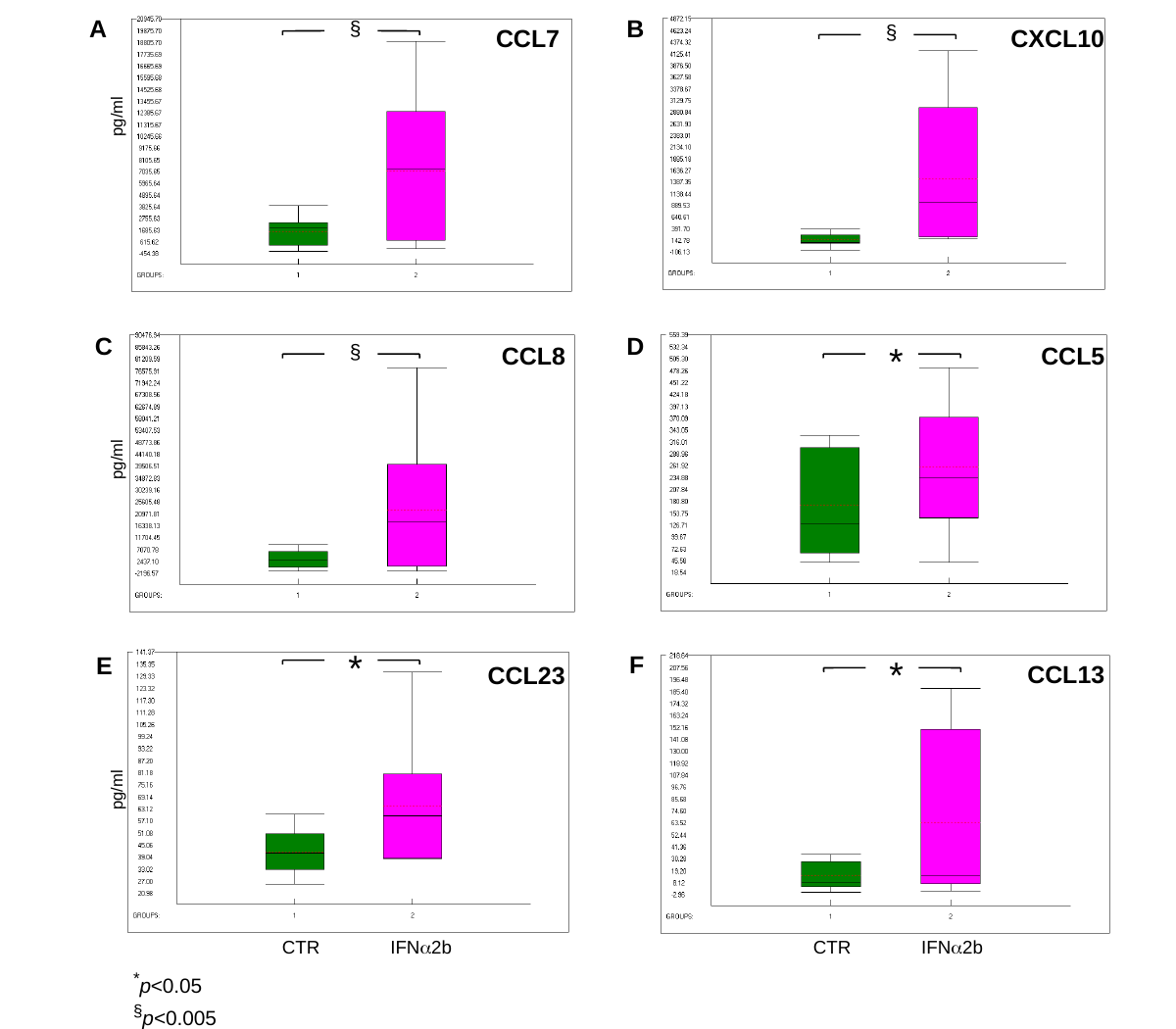

A
B
§
§
CCL7
CXCL10
CCL5
CCL8
CCL13
CCL23
pg/ml
D
§
*
pg/ml
*
F
*
pg/ml
CTR
IFNα2b
CTR
IFNα2b
*p<0.05
§p<0.005
C
E

Supplement: Additional file 3 — Release of chemotactic chemokines by CD14+ monocytes exposed to IFNα in vitro. Chemotactic chemokines released by monocytes isolated from healthy donors and exposed in vitro to 103IU/ml of IFNα. The graph shows the 6 factors selected, out of a panel of 46 tested, for being significantly enriched in the supernatants of cells exposed to the cytokine as compared to untreated controls. The box plot graph shows for 10 samples per group: Red line: Mean, Black line: Median, Box: 25th to 75th percentile, whiskers:10th to 90th percentile. *p < 0,005, §p < 0,05 (Wilcoxon Matched Pairs test). [file 1479-5876-9-67-S3.PPT]
